# Automatic quantitative analysis of structure parameters in the growth cycle of artificial skin using optical coherence tomography

**DOI:** 10.1117/1.JBO.26.9.095001

**Published:** 2021-09-01

**Authors:** Ruihang Zhao, Han Tang, Chen Xu, Yakun Ge, Ling Wang, Mingen Xu

**Affiliations:** a Hangzhou Dianzi University, School of Automation, Hangzhou, China; b Key Laboratory of Medical Information and 3D Bioprinting of Zhejiang Province, Hangzhou, China

**Keywords:** optical coherence tomography, artificial skin, roughness model, adaptive interface detection

## Abstract

**Significance:** Artificial skin (AS) is widely used in dermatology, pharmacology, and toxicology, and has great potential in transplant medicine, burn wound care, and chronic wound treatment. There is a great demand for high-quality AS product and a non-invasive detection method is highly desirable.

**Aim:** To quantify the constructure parameters (i.e., thickness and surface roughness) of AS samples in the culture cycle and explore the growth regularities using optical coherent tomography (OCT).

**Approach:** An adaptive interface detection algorithm is developed to recognize surface points in each A-scan, offering a rapid method to calculate parameters without constructing OCT B-scan pictures and further achieving realizing real-time quantification of AS thickness and surface roughness. Experiments on standard roughness plates and H&E-staining microscopy were performed as a verification.

**Results:** As applied on the whole cycle of AS culture, our method’s results show that during the air–liquid culture, the surface roughness of the skin first decreases and then exhibits an increase, which implies coincidence with the degree of keratinization under a microscope. And normal and typical abnormal samples can be differentiated by thickness and roughness parameters during the culture cycle.

**Conclusions:** The adaptive interface detection algorithm is suitable for high-sensitivity, fast detection, and quantification of the interface with layered characteristic tissues, and can be used for non-destructive detection of the growth regularity of AS sample thickness and roughness during the culture cycle.

## 1 Introduction

Engineering of artificial tissue and organs is a realm of significant activity in biotechnological and biomedical research.^1^^–^^3^ Among all the organs in the human body, the skin is the largest and serves as a protective barrier against several environment hazards. When the skin is wounded by trauma, injury, or skin disease, this may lead to the loss of protective functions and can cause even worse problems. As an equivalent or replacement for human skin, artificial skin (AS) has the ability to serve as the fast treatment for such injury.^4^ In addition, AS is of great importance for cosmetics research and testing. In recent years, with the accelerated innovation of anti-aging skin care products and the development of more effective skin products, it is estimated that by 2021, the global anti-aging cosmetics market value will reach 331.4 billion US dollars.^5^ The cosmetics industry requires a large amount of experimental research, but stricter laws have been enforced against animal experiments [e.g., the implementation of European Directive 2003/15/EC2(1)].^6^ As a result, AS is becoming a promising substitute for animal skin in such experiments. Moreover, AS is widely used in dermatology, pharmacology, and toxicology and has great potential in transplant medicine, burn wound care, and chronic wound treatment.^1^^,^^3^^,^^7^^–^^9^ To meet the demand of all the application fields, it is vital to achieve a large amount of high-quality AS preparation.

At present, among different AS preparation methods, air–liquid culture is the most popular, in which keratinocytes are inoculated on the collagen matrix layer and diffused into multiple layers to build the AS.^10^ During the culture cycle, the cells begin to proliferate and form a layer of stratum corneum. However, due to the natural fluctuations in the growth of cell organisms and the dependence of biological cells factors such as the age of the donor, cell density, and cell viability, a monitoring method is necessary to measure the quality and condition of the AS.^2^^,^^3^^,^^7^ The quality and performance of AS can be determined by structural parameters. One factor that causes failure in AS preparation is exudation of the culture medium; this causes a distinct, curved surface under optical coherent tomography (OCT), which can be recognized by the thickness distribution analysis of the AS sample. Thickness reflects the growth state of AS during preparation, and the average thickness is usually a criterion for quality. Skin roughness is another important structural parameter in medical diagnosis and evidence reports on cosmetics, used to provide reliable feedback on skin structure and growth.^5^^,^^11^^,^^12^ Therefore, the question of how to evaluate the structural parameters in the AS growth cycle, non-invasively and effectively, is of great significance. Considering the large number of AS samples to be measured in mass production condition and to avoid the influence of measuring operation on the culture process, real-time measurement is highly favored.

Currently, many methods have been reported to measure real skin or AS structure parameters, such as confocal microscopy, PRIMOS, fluorescence microscopy, OCT, and second-harmonic microscopy.^13^^–^^16^ Among them, OCT is a promising technique that can realize non-invasive, real-time three-dimensional (3D) imaging in the order of micrometers in biological tissues, and can detect structural information of samples.^17^^–^^21^ Askaruly et al.^5^ performed skin boundary recognition on OCT images and calculated skin roughness based on the definition of the ISO 25178-part 2 standard. By comparing with the results of PRIMOS skin measurement equipment, it can be considered that the 3D volume and depth imaging capabilities of OCT can reduce image artifacts, demonstrating the potential of OCT for providing reliable and quantitative skin surface roughness. However, the above-mentioned research did not offer a quantitative assessment of the skin roughness. Schmitt et al.^8^ sampled the upper surface of the AS at large intervals using OCT to characterize macroscopic surface tears, defect morphology, and microscopic cell keratinization of the skin. Owing to the limitation of sampling accuracy in the interval sampling, the 2D data can only reflect local features, thus failing to quantify skin roughness. Schmit et al.^1^ used OCT to monitor the growth cycle of AS, and OCT tomograms were taken after each production step of the skin equivalents and compared with the histological images, but lacked the quantification of structural parameters. Gambichler et al.^22^ calculated the epidermal thickness of the skin through the characteristic peaks of the OCT intensity signal of the skin epidermis, which provided a theoretical basis for quantifying the upper and lower surfaces of the skin based on the OCT intensity signal. However, the overall thickness is represented by the thickness of the A-scan at several better positions, which has high subjectivity and uncertainty. Kepp et al.^23^ used the convolutional neural network to segment different layers of mouse skin and measured the thickness of each single skin layer. However, such machine learning-based methods need a large training set for 3D images, which requires a lot of calculation time, and the quantification is still needed after the segmentation.

As mentioned above, many studies have reported the use of OCT to evaluate the thickness or roughness of skin, but fast quantification without human interference is still not achieved. One major obstacle is the noisy signal of OCT, which often causes speckles at the surface, making conventional algorithms such as the binarization method fails to find the true interface. In this study, we optimized our previously proposed method for detecting the thickness of 3D-printed skin^24^ and developed a fully automatic adaptive interface detection algorithm to find the AS surface, overcoming the noise problem. As interface points (i.e., upper and lower surface points) in each A-scan are obtained from signal analysis, parameters can be calculated even without construction of B-scan and 3D pictures, which reduces the operation time and improves the quantification efficiency. Structural parameters such as thickness and roughness were quantified through the AS surface contour calculation. Because the proposed method is real-time, non-invasive, and free of human interference, it is a promising method for AS quality monitoring under mass production conditions.

## 2 Method

### 2.1 Materials and Methods

We adopted the Skinovo AS model (Hangzhou Regenovo Biotechnology, Ltd.), in which keratinocytes are used as seed cells and printed on the transwells (Corning lnc., 3413) for air–liquid culture. AS samples were formed through the process of proliferation and differentiation, and air–liquid cultured in a serum-free medium for maturation. A standard culture cycle has 13 days and the cellular differentiation usually begins on day 5;^24^ therefore, days 1, 5, 9, and 13 were selected for testing to obtain roughness at different culture stages. Data of the AS sample were collected through OCT, and then the surface parameters were obtained using the proposed algorithm. To describe the overall structure of the AS, OCT data with a size of 9  mm×9  mm×3.59  mm (1000  pixels×1000  pixels×1024  pixels) in the whole AS sample were collected. In this experiment, five AS samples were continuously monitored as batch 1 to observe the change in surface morphology with the culture time, and another two batches (i.e., batches 2 and 3) with five samples each were arranged for analysis between batches. Batch 4 with four AS samples was used for H&E staining microscopy test, and batch 5 with four AS samples was a collection of abnormal skin samples.

We used a self-developed spectrum-domain OCT system based on a Michelson interferometer configuration.^24^ The system uses a broadband light source with a central wavelength of 1310 nm and a full width at half maximum of 248 nm. In the actual measurement, the system has an axial resolution of 3.5  μm, a lateral resolution of 13  μm, and an imaging depth of 3.59 mm in air. The refractive index of the entire AS was 1.38, yielding an axial resolution of 2.53  μm in AS. The A-scan rate is 48 kHz. In our OCT setup, the optical path difference and focus position relative to the AS sample were fixed in all experiments.

### 2.2 Adaptive Interface Detection Algorithm

**Fig. 1:**
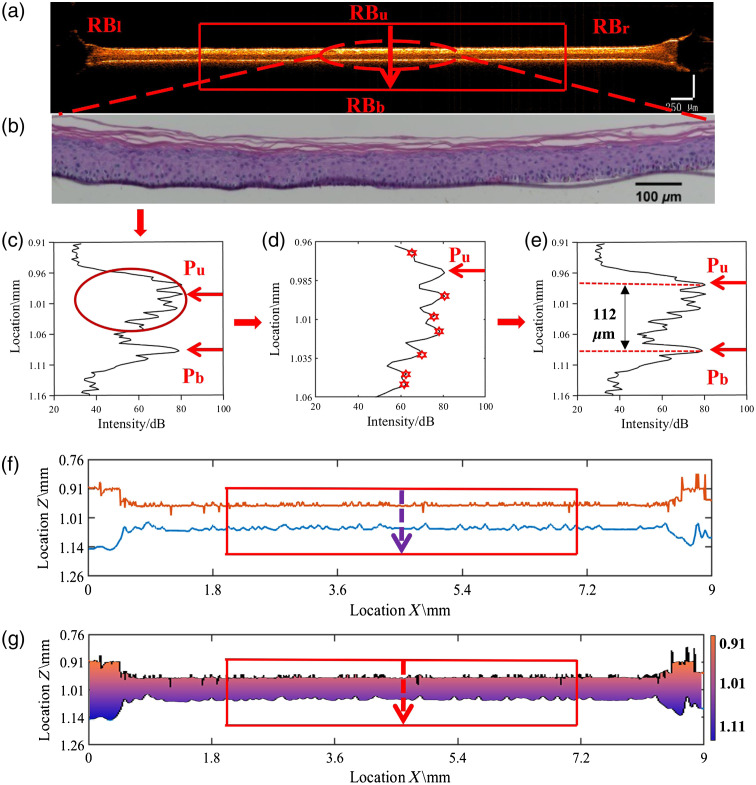
Flowchart of the adaptive interface detection algorithm. (a) Calculation of the lateral boundary value, i.e., $RB_{l}-RB_{r}$ of AS based on the drop of the peak of the skin boundary, where the effective longitudinal range of the sample $RB_{u}-RB_{b}$ is set according to the cross-sectional view. (b) H&E-stained image of AS structure, and it shows good consistency with the thickness value obtained by the algorithm. (c) Positioning of the upper and lower surfaces of each A-scan of AS. (d) Repositioning of the upper and lower surface points of the skin sample according to the local maximum points in the range (local zoomed). (e) Accurate positioning of top and bottom surface points. (f) Extraction of the upper and lower AS surface points. (g) Calculation of roughness and thickness based on the AS surface points, where different colors represent different heights.

Accurate positioning of the surface is key to quantifying the structural parameters of the AS. Figure 1 shows a typical B-scan image of AS under OCT and two A-scan signals at different positions. The air–AS interface causes a peak in the signal, which helps to locate the surface. However, owing to the influence of environmental noise and biological structure, there will be false peaks or burrs in the OCT signal in the A-scan. In some cases, multiple signal peaks can be detected in the interface attachment, causing the conventional peak detection method to fail, and thus requiring human interference. To achieve high efficiency by avoiding manual operations or judgement, an adaptive algorithm is developed. The algorithm controls the detection time of the upper and lower interfaces of the entire 3D AS sample within 45 s, which provides the possibility for real-time monitoring. The computer uses i5-5200U CPU, M250 graphics card, and R2019a version of MATLAB.

The proposed algorithm is fulfilled in MATLAB, which takes the signal data cube captured by OCT as the input and the structural parameters (i.e., thickness and roughness values) as the output. The automatic processing steps of the automatic algorithm are as follows.

1. *ROI box determination and background removal*. Because the relative height of the focal point of OCT is fixed, which can easily be guaranteed by integrating an OCT system with a certain AS preparation pipeline, the height region of interest (ROI) is realized by intercepting a calibrated range in the A-scan signal that contains the AS. The width of the ROI is predetermined by the diameter of the AS samples. Signals outside the ROI were removed to exclude unwanted noise, as shown in Fig. 1(a). The four margins of the ROI box are marked as $RB_{u}$, $RB_{b}$, $RB_{l}$, and $RB_{r}$, respectively.
2. *A-scan data preparation*. For the 3D AS sample, the ROI is a cylinder with a certain center and radius. A-scan signals within the ROI are extracted to form a data matrix for further processing. In our AS parameter monitoring setup, the ROI has a radius of 2.5 mm on the AS surface.
3. *Coarse recognition of AS interface*. The peak with the highest intensity signal value of each A-scan is obtained using the maximum function. This position is marked as an index. The position of a second peak with the highest intensity signal for the rest part is searched in the same way, and the pixel pitch of these peaks is used to determine whether it is less than the predetermined threshold T1. The loop breaks and the positions of these peaks are determined (i.e., $P_{u}$ and $P_{b}$) if it is satisfied. Otherwise, the above process will be continued, as shown in Fig. 1(c). Because the minimum thickness of the AS is approximately 25  μm on day 1, the T1 value is 10 pixels and is used to eliminate the effect of noise and ensure that all peaks are accurately identified. For the same reason, the T1 value is 15 pixels on day 9 and 20 pixels on day 13.
4. *Fine recognition of AS interface*. The upper surface of the skin sample was re-identified in this step. Due to the high degree of cell keratinization in the middle and late stages of culture, a multilayered keratinous structure will be formed gradually, and the A-scan signal of the stratum corneum will have multiple spikes and small fluctuations in the condition, and the base membrane will also interfere with the detection of the lower surface. Therefore, the signal searching range on the upper surface is refined to [$RB_{u}$, $(P_{u}+P_{b})/2$], and the signal searching range on the lower surface is refined to [$(P_{u}+P_{b})/2$, $RB_{b}$]. All the maximum points in the signal range are recorded, numbered, and marked as peak(i). If the difference between the intensity value of the maximum point peak(i) and the intensity value of $P_{u}$ or $P_{b}$ is greater than the threshold T2, then the MATLAB program will continue to compare it with the next maximum point until they are all compared. Otherwise, if the difference is less than the fluctuation error threshold T2, the point above the physical position is chosen as the true surface of the AS, and the position is marked as the new $P_{u}$ or. $P_{b}$ As shown in Fig. 1(d), the frequency statistics of the ratio between the intensity signal of the actual surface peak $P_{u}$ or $P_{b}$ and the coarse result identified by the algorithm show that the peak resulting from the multi-samples is mostly within 20%; therefore, 20% of the $P_{u}$ or $P_{b}$ signal is set as T2.
5. *Structural parameter quantification*. As shown in Fig. 1(e), the vertical pixel positions of the upper and lower surfaces of the AS samples are extracted and saved to extract the AS interface. As detailed in Sec. 2.3, the thickness and roughness are quantified based on the AS interface.

### 2.3 AS Parameter Quantification

The thickness is obtained by the difference between $P_{u}$ and. $P_{b}$ The actual thickness between the peaks is calculated according to the refractive index:

$$Th=\frac{\delta }{\gamma }\times N.$$ (1)

In the above equation, δ and γ represent the pixel resolution and the refractive index of the skin, respectively, and N is the number of pixels between the peaks in the intensity signal of the A-scan.

Roughness is another important feature that can reflect the irregular shape of the skin surface. For AS, keratinization of the cells will cause changes in the keratinous structure, which can vary the value or pattern of surface roughness. In this study, after the surface point cloud is obtained by the adaptive algorithm, the skin surface structural morphology changes are analyzed by calibrating the skin surface points. Owing to the possibility of sample tilting or natural skin growth during the AS culture period resulting in an uneven skin surface, this study used binary cubic surface fitting to flatten the skin surface before calculating the roughness.

For a well-cultured AS sample, the upper layer is the corneum formed in the culture cycle and is of vital importance to the protection ability of AS. The roughness evaluation of AS is a direct evaluation of the corneum. Thus, we chose two aspects of roughness, namely the magnitude of roughness and the distribution pattern of surface height.

The International Organization for Standardization provides us with several criteria that characterize the degree of roughness, from which the average roughness is adopted in this study to express the magnitude of AS roughness.^5^ Equation (2) defines the definition of the average roughness $R_{a}$:

$$R_{a}=\frac{1}{PN}\times \overset{{\left(x_{n}-x_{c}\right)}^{2}+{\left(y_{n}-y_{c}\right)}^{2}<r^{2}}{\underset{n}{\sum }}|Z_{n}-\overset{¯}{Z}|,$$ (2)

where $\left(x_{c},y_{c}\right)$ is the center position ROI of the skin, r is the radius of ROI, $\left(x_{n},y_{n},Z_{n}\right)$ is the coordinate of the n’th sampled point, $\overset{¯}{Z}$ is the average surface height in the ROI, and PN is the number of sampling points in ROI, respectively.

In addition to $R_{a}$, another criterion, $R_{sk}$, quantifies the bias distribution of the surface height. And to obtain $R_{sk}$, the root-mean-square roughness, $R_{q}$, needs to be calculated first. The expressions for $R_{q}$ and $R_{sk}$ are defined in Eqs. (3) and (4), respectively:

$$R_{q}=\sqrt{\frac{1}{PN}\times \overset{{\left(x_{n}-x_{c}\right)}^{2}+({y_{n}-y_{c})}^{2}<r^{2}}{\underset{n}{\sum }}{\left|Z_{n}-\overset{¯}{Z}\right|}^{2}},$$ (3)

$$R_{sk}=\frac{1}{R_{q}^{3}}\times \frac{1}{PN}\times \overset{{\left(x_{n}-x_{c}\right)}^{2}+{\left(y_{n}-y_{c}\right)}^{2}<r^{2}}{\underset{n}{\sum }}{\left(Z_{n}-\overset{¯}{Z}\right)}^{3},$$ (4)

where PN, $x_{c}$, $y_{c}$, $\overset{¯}{x}$, $\overset{¯}{y}$, ($x_{n}$, $y_{n}$, $Z_{n}$), and r have the same meaning as in Eq. (2). $R_{sk}$ reflects the symmetry property of the surface roughness. When the value of $R_{sk}$ is closer to 0, the height distribution of the sample surface is more symmetrical. The peak roughness is closer to the average surface of roughness.

## 3 Verification Experiment

To demonstrate the accuracy and feasibility of our roughness evaluation method, four roughness plates were tested, and the results were compared with the algorithm^5^ and the standard values of the model. Four roughness plates (vertical milling roughness model from Weifang Huaguang Measuring Tool Co., Ltd.) that meet the Chinese National Standard were used. Their $R_{a}$ values are 1, 1.6, 3.2, and 5.8  μm, respectively.

In the verification experiment, the focus of the OCT acquisition was positioned on the surface of the roughness model, and then nine patches of 5  mm×5  mm×2.59  mm (556  pixels×556  pixels×1024  pixels) 3D OCT data were collected, and then the proposed algorithm was applied to obtain its arithmetic mean roughness.

**Fig. 2:**
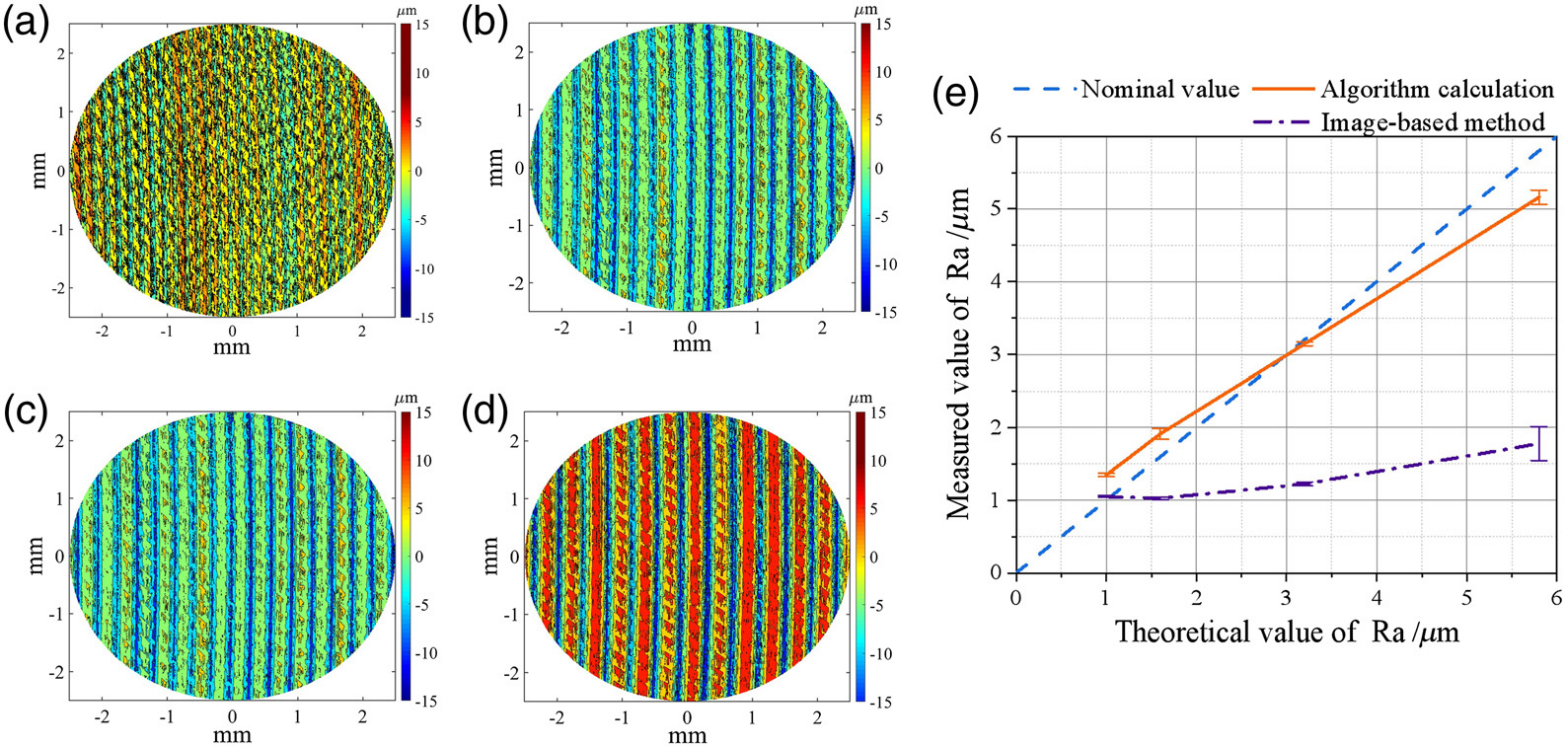
Roughness model contour map and roughness verification curve. (a) $R_{a}=1.0\;\mu m$, (b) $R_{a}=1.6\;\mu m$, (c) $R_{a}=3.2\;\mu m$, (d) $R_{a}=5.8\;\mu m$, and (e) verification curves of our algorithm and the Askaruly’s Method.^5^

The surface roughness was calculated according to the definition in Sec. 2.3, and the results are illustrated in Figs. 2(a)–2(d). The verification curve of the roughness algorithm is shown in the Fig. 2(e), and all the data are listed in Table 1.

**Table 1:** Roughness verification results.

Arithmetic mean roughness	Model number			
	1	2	3	4
Nominal value (μm)	1.0	1.6	3.2	5.8
Algorithm result (μm)	1.35 ± 0.02	1.92 ± 0.07	3.14 ±0.03	5.16 ±0.10
Image-based method^5^ (μm)	1.05 ± 0.01	1.03 ± 0.02	1.22 ± 0.03	1.78 ± 0.23

Figures 2(a)–2(d) show the surface contour maps of the roughness plate when $R_{a}$ is 1, 1.6, 3.2, and 5.8  μm. In these figures, the average surface height is defined as zero. Figure 2(e) shows the comparison between the proposed algorithm and the image-based method.^5^ The horizontal and the vertical axes denote the true value and the measured value, respectively, and the dashed blue line has the slope of 1, which is the no-error condition. It can be noted that the slope of the verication curve of the proposed algorithm is 5.2 times greater than the image-based method, showing a much higher sensitivity in roughness measuring. The image-based method’s low sensitivity leads to the failure to effectively distinguish the roughness value in the range between $R_{a}=1$ and 1.6  μm, and this curve shows greater deviation for high $R_{a}$ conditions. For our proposed adaptive method, which is based on the intensity signal, the maximum deviation from true value is 0.64  μm. Since this maximum deviation occurs at the highest $R_{a}$ condition, relative error is only about 11%. Even through the deviation, the value of the algorithm obtained by the adaptive interface detection algorithm shows a positive, linear relation with the nominal value of the roughness model, and the variance of the tested value for each sample plate remains small. Thus, the results imply the capability of the proposed method to differentiate AS of different Ra levels on a micrometer scale.

## 4 Artificial Skin Quantification

The structural parameters of the three batches of AS samples were monitored during the air–liquid culture cycle. The AS samples were tested on days 1, 5, 9, and 13. Statistical analysis based on roughness measurement was performed to study the surface morphology change with the culture time. A batch of typical failure samples were specially customized, and their thickness and roughness parameters were analyzed.

### 4.1 Quantitative Analysis of Thickness and Roughness of Normal Skin Growth Cycle

**Fig. 3:**
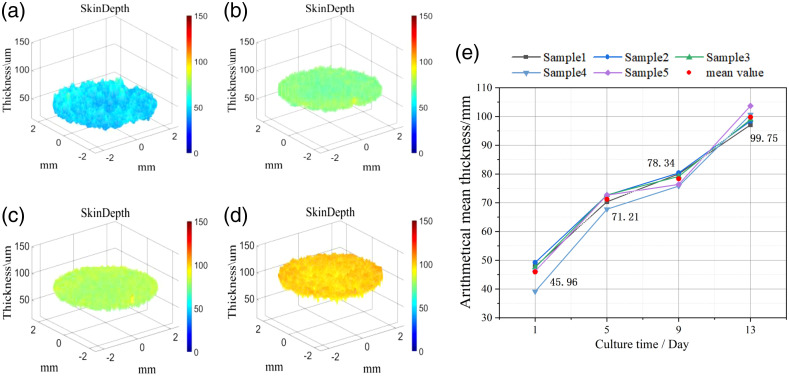
Thickness distribution and thickness curve of AS at different culture times. (a) Day 1, (b) day 5, (c) day 9, and (d) day 13. (e) The Th curves of five AS samples during the culture period.

Figures 3(a)–3(d) show the distribution of the thickness of the same AS on days 1, 5, 9, and 13 obtained by the structure parameter monitoring method in Sec. 2. From days 1 to 5, the distribution range of the overall skin thickness is reduced, the thickness growth rate is larger, and the structure tends to be flat. From day 5 to 13, the thickness of the AS samples grew slowly, the surface gradually became rougher, and the overall thickness growth rate increased on day 13. The statistical average thickness of the five AS samples in batch 1 are obtained, and the results are shown in Fig. 3(e). The Th values of different samples maintain a high degree of similarity, and the thickness increases with the air–liquid culture time. The sample has a larger increase on the days 5 and 13.

**Fig. 4:**
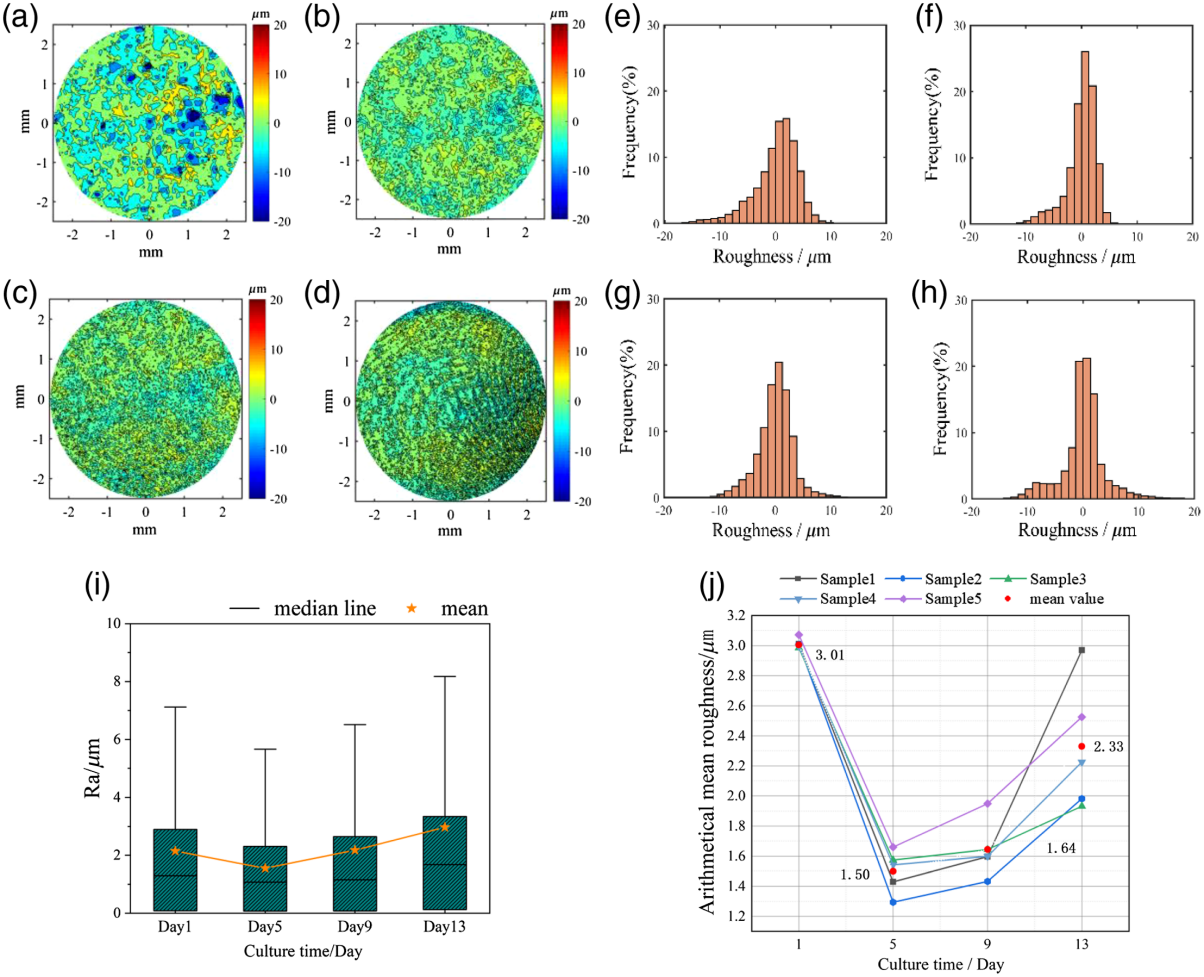
Surface contour maps, frequency histogram, box plot, and $R_{a}$ curve of AS at different culture times. (a)–(d) The surface contour maps during the culture period; (e)–(h) the frequency histograms during the culture period; (i) the box plots of a single sample; and (j) the $R_{a}$ curves of five AS samples during the culture period.

The distribution of the surface height during the culture cycle is a method of roughness evaluation, as shown in Figs. 4(a)–4(d). On day 1, hilly conditions on the surface existed, which contributed to the high value of $R_{a}$. This hilly surface shape was mainly due to the cell bumps that were unevenly distributed in the inoculation. Compared with day 1, the size of each peak or valley in the contour map becomes smaller and more evenly distributed with culture time. During the culture process from days 1 to 5, the AS surface tended to get flat. In the subsequent air–liquid culture process from days 9 to 13, the scattered and dot-like protruding areas on the skin surface were gradually connected into blocks, and the fluctuations on the skin surface show obvious regional characteristics.

To further analyze the distribution of the roughness of AS in different stages, the relative surface height frequency histogram of the entire cultivation cycle of the same batch of AS samples is shown in Figs. 4(e)–4(h). In this figure, the average surface height is defined as zero. On day 1 [Fig. 4(e)], the distribution of surface height was mainly in the range of −15 to 10  μm on the average surface of the AS, and the distribution is relatively loose. On day 5 [Fig. 4(f)], the relative height distribution was more concentrated, with a distribution mainly between −10 and 5  μm. From days 5 to 13 [Figs. 4(g) and 4(h)], the distribution range of the surface increases. From the box plots in Fig. 4(i), it can be seen that the median, upper quartile, and upper limit of roughness show a trend of first decreasing and then increasing in the course of cultivation time.

The statistical average roughness of the five AS samples in batch 1 are obtained, and the results are shown in Fig. 4(j). In terms of roughness parameters, the sample had comparable $R_{a}$ values on day 1 when standard inoculation was performed. As time increased, the $R_{a}$ curves varied, which is mainly due to the difference in cell growth activity between samples. The values of $R_{a}$ at the end of the culture cycle show a relatively large difference, that is, varying from 1.9 to 3  μm. Despite this difference, all the $R_{a}$ curves of the AS samples show first a drop and then an increase during the process of air–liquid culture. The average $R_{a}$ reached a minimum on day 5 and then gradually increased with the time of air–liquid culture. $R_{sk}$ also showed a decrease from days 1 to 5 and an increase from days 9 to 13 for all the AS samples.

The statistical table of changes in the structural-related parameters of different batches with the air–liquid culture time is shown in Table 2. For the thickness parameter, all batches increased with the increase in incubation time but at a different rate between different batches. For the roughness parameter, the roughness difference between batches on day 1 can be explained by the printing condition. Due to the difference in cell activity between batches, the values of the roughness parameter also show notable differences on day 13. All the batches share the same trend for $R_{a}$ and $R_{sk}$.

**Table 2:** AS structure parameter changes of different batches.

Batch	Roughness parameter	Culture time/day			
		1	5	9	13
1	Th (μm)	45.96 ± 3.96	71.21 ± 2.12	78.34 ± 2.17	99.75 ± 2.54
	$R_{a}$ (μm)	3.01 ± 0.04	1.50 ± 0.14	1.64 ± 0.19	2.33 ± 0.43
	$R_{sk}$	2.92 ± 0.19	2.17 ± 0.22	2.49 ± 0.10	3.32 ± 0.33
2	Th (μm)	37.82 ± 5.21	45.54 ± 2.76	66.24 ± 3.44	80.46 ± 4.20
	$R_{a}$ (μm)	3.06 ± 0.38	1.65 ± 0.23	1.96 ± 0.22	2.62 ± 0.20
	$R_{sk}$	2.79 ± 0.42	2.33 ± 0.16	2.64 ± 0.32	2.85 ± 0.30
3	Th (μm)	37.08 ± 1.74	47.62 ± 1.17	61.45 ± 1.12	75.65 ± 0.86
	$R_{a}$ (μm)	2.97 ± 0.13	1.82 ± 0.18	2.23 ± 0.38	2.31 ± 0.24
	$R_{sk}$	2.68 ± 0.44	2.11 ± 0.16	2.14 ± 0.18	2.43 ± 0.12

To verify the feasibility of our method, we performed repeated measures of multi-factor analysis of variance^25^ to statistically analyze the $R_{a}$ and Th data in Table 2, as shown in the Supplemental Material. Considering the difference in thickness at the starting point, the increase rate of Th is adopted for analysis. Statistical analysis with time is performed, and we obtain F−Th−rate (3, 10) = 77.019 (P=3.2677×10−7<0.05) and $F-R_{a}(3,36)=85.272$ (P=2.0111×10−16<0.05). The results indicate that Th and $R_{a}$ have significant statistical differences with time, which show that the culture stage can distinguished by such parameter monitoring. The results of statistical analysis between batches show that F−Th−rate (2, 12) = 0.913 (P=0.427>0.05) and $F-R_{a}$ (2, 12) = 3.107 (P=0.082>0.05), which means the value of $R_{a}$ and the increase rate of Th show no significant statistical difference between batches.

### 4.2 Comparison with H&E Staining

**Fig. 5:**
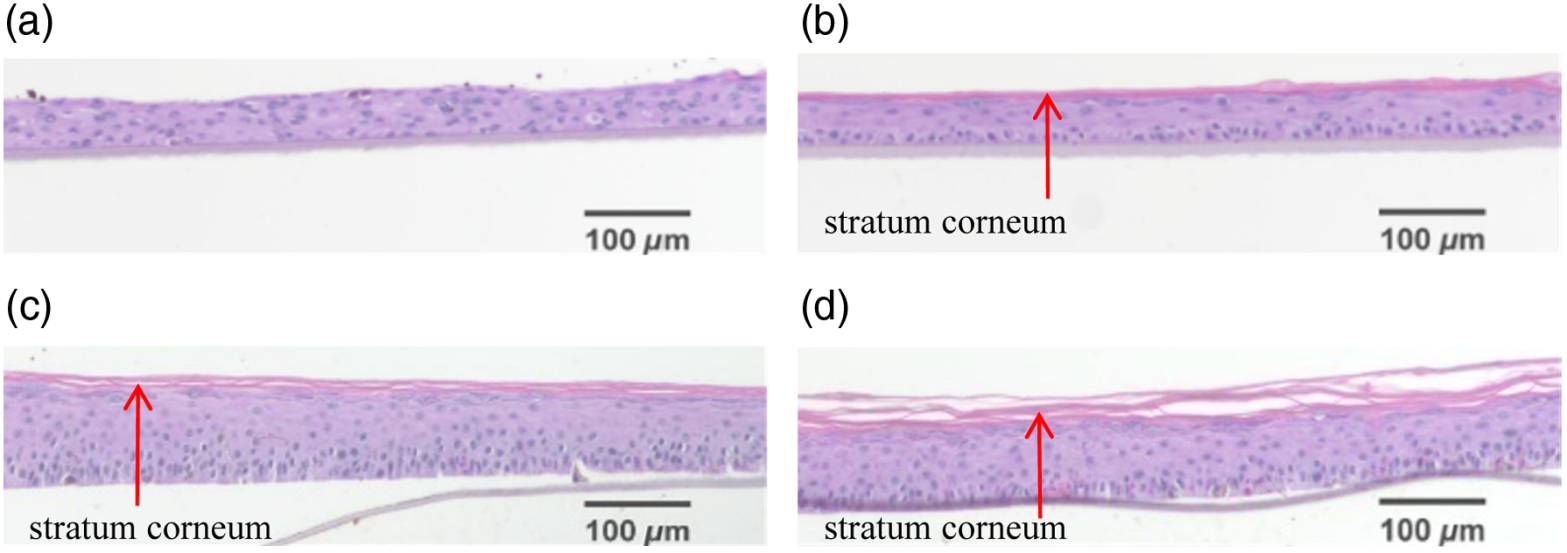
Images of AS samples under a microscope after H&E staining. (a)–(d) H&E staining of AS under the 10× microscope on days 1, 5, 9, and 13, respectively. The corneum part and the rest of the AS can be distinguished by the difference in color. It shows qualitatively that the overall thickness increased with time, with no sign of the corneum on day 1, and from days 5 to 13, the thickness of the corneum layer continues to increase. From panels (a) and (b), it can be seen that the unevenness caused by inoculation disappeared in the culture cycle.

To show the condition of the stratum corneum during the culture cycle, four AS samples of batch 4 were stained and cut for microscopy observation on days 1, 5, 9, and 13. Before cutting, the AS was fixed with a 4% formaldehyde solution for 24 hours, and then the skin slices were made by dehydration, embedding, and H&E staining. Under microscope, the corneum along with the rest of AS can be distinguished by the difference in color and shape, as shown in Fig. 5. No obvious corneum was observed on day 1, and on day 5, a thin layer of stratum corneum was observed. From days 5 to 13, the degree of fluctuation of the stratum corneum increased, which is in agreement with the increase of Th in this period. The undulating surface caused by the inoculation operation on day 1 became less notable on day 5, which is in agreement with the decrease of $R_{a}$. Thus, it is reasonable to draw a relationship between the $R_{a}$ value and the stage of culture, especially associated with the corneum condition. Considering the relative height distribution in Fig. 4(i), the histogram of relative height can also reflect the corneum condition, and the increase in H&E slice thickness is consistent with the increase in thickness value.

### 4.3 Parameter Analysis of Typical Failure Conditions

**Fig. 6:**
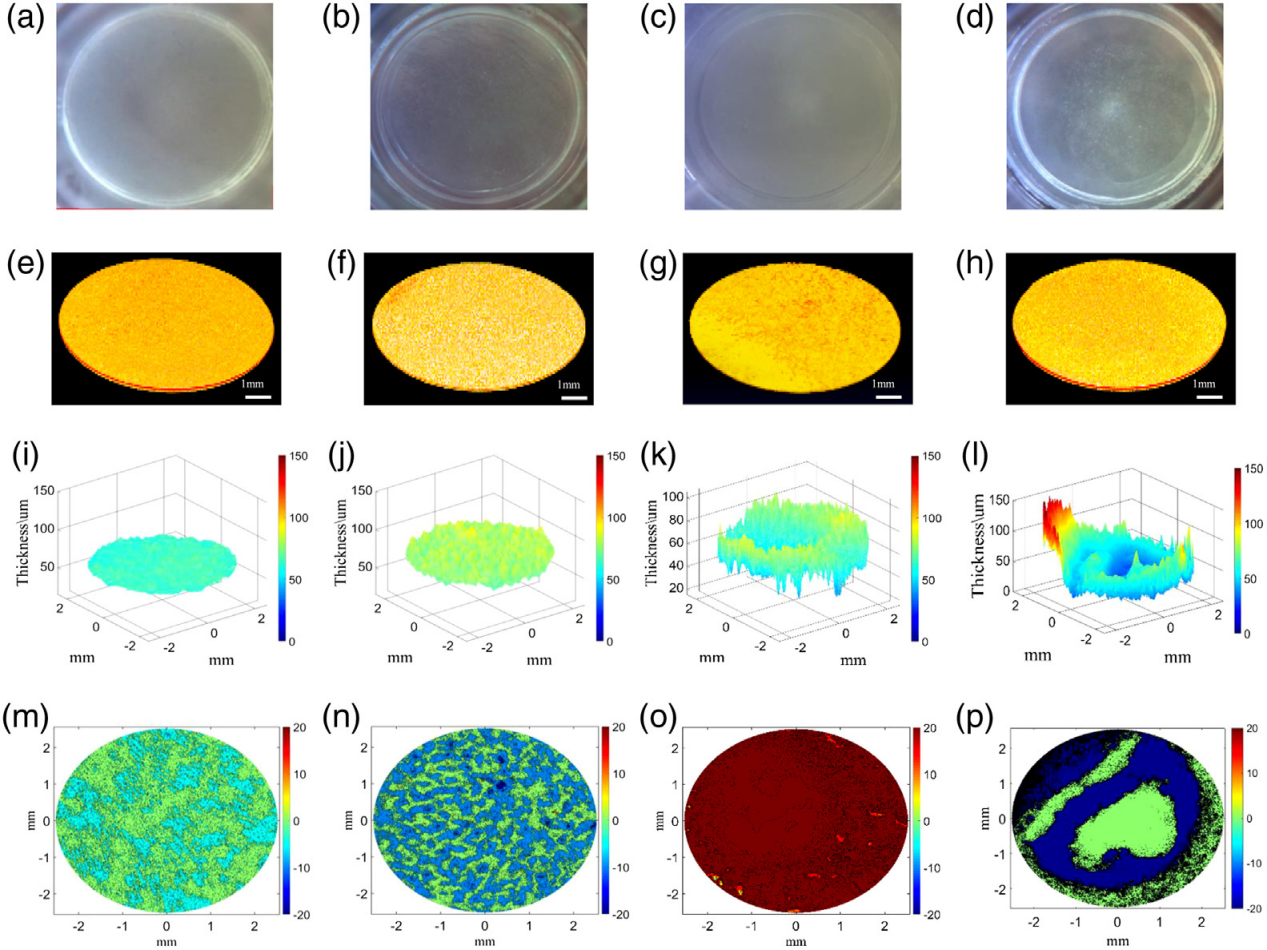
The images, thickness, and surface contour maps for one normal sample and three abnormal samples. (a)–(d) The images of the normal sample, the abnormal-shaped sample, the sample with exudation, and the sample with cuticle exfoliation, respectively. (e)–(h) The 3D simulation diagrams of four samples. (i)–(l) The thickness maps for the four samples, and (m)–(p) the surface contours for the four samples.

Unexpected abnormal samples may occur, even though the culture environment and the process are strictly controlled. To study the changes in the thickness and roughness of AS samples under different failure conditions, we specially customized a batch of failed skin samples as batch 5, including the most common abnormalities such as surface gully, exudate, and stratum corneum peeling. Figure 6 shows the photographic images, 3D reconstruction pictures, thickness maps, and roughness distribution maps of a normal sample and three typical abnormal samples on day 7 of culture.

Figures 6(a)–6(d) show the images of the normal sample, the surface gully sample, the exudate sample, and the stratum corneum exfoliation sample; Figs. 6(e)–6(h) show the surface maps after the 3D data are reconstructed. Figures 6(i)–6(l) show the thickness distribution diagrams of the samples; Figs. 6(m)–6(p) show the roughness distribution maps of the samples. It can be seen from Fig. 6 that it is difficult to distinguish abnormal samples from normal samples by photographic images, and it is also difficult to observe particularly obvious differences from reconstruction images, but the thickness and roughness data may reveal this information. Therefore, after quantitative processing, the average thickness of the three abnormal samples are 49.4, 103, and 55.9  μm while the average thickness of the normal sample is 46.7  μm. From the thickness distribution diagram, the thickness distribution of the abnormal sample is uneven, and there are a lot of raised and gully. The average roughness ($R_{a}$) of the three abnormal samples are 7.2, 8.66, and 17.3  μm while the normal $R_{a}$ is 2.3  μm. Therefore, the roughness of the abnormal samples has a relatively large increase. The results show that the thickness and roughness data of normal and abnormal samples are significantly different.

## 5 Discussion

This study uses OCT to evaluate AS structural parameters (i.e., thickness and roughness), where an automatic peak detection algorithm is developed to accurately extract the skin surface through the 3D data of each AS, after which thickness and roughness parameters are calculated. Verification of thickness was achieved with microscopy images, and experiments on standard roughness plates demonstrated the feasibility of the proposed method when under in low-roughness conditions ($1\;\mu m<R_{a}<5.8\;\mu m$). Considering the measured average $R_{a}$ of AS in each batch during the culture cycle ($1.5\;\mu m<R_{a}<3.06\;\mu m$), the feasibility range of our proposed method covers the roughness range of AS samples during the culture cycle.

For the observation of AS during the culture cycle, the results show that the surface distribution of the skin varies greatly in different air–liquid culture stages. At the beginning of the AS air–liquid culture (day 1), when the cells were not been keratinized, the uneven surface of the AS was caused by the distribution of cell clusters [Figs. 5(a) and 4(a)]. The skin presents irregular protrusions, as shown in Fig. 4(e) and Table 2. More protrusions indicated that the structure was loose, and the roughness of different positions varied greatly, resulting in larger $R_{a}$ values, and the asymmetry of the roughness frequency distribution results in a large $R_{sk}$ value. From days 1 to 5 in air–liquid culture, the degree of cell keratinization remains low, and the skin structure was smoother and firmer [Figs. 4(b) and 5(b)]. During this period, the value of $R_{a}$ drops sharply, and the skin structure is firmest. It shows that the AS mainly promotes formations of the basal layer form days 1 to 5. The epidermal structure is closely connected, the skin surface is the flattest [Fig. 4(f) and Table 2], and the frequency distribution is the most symmetrical, leading to the minimum $R_{sk}$. As the air–liquid culture reached day 9, with the increase and differentiation of keratinocytes, the stratum corneum clearly showed a layered structure, and the scattered protrusions on the AS surface indicate that the degree of keratinization has increased [Figs. 4(c) and 5(c)], with the structure of the skin gradually roughened. The amplitude of surface surface fluctuations gradually increased, leading to a likewise gradual increase in $R_{a}$. As the proportion of surface convexity in the roughness frequency distribution increased, the surface light was scattered by point-like convexity. It has little effect on the overall smoothness of the skin surface [Fig. 4(g) and Table 2], leading to only a slight increase in $R_{sk}$. When the air–liquid culture was carried out on day 13, the thickness increased and the stratum corneum of the AS was more differentiated. The scattered and dot-like protruding areas on the surface gradually show the characteristics of local massive tight connections [Figs. 4(d) and 5(d)], the large increase in the amplitude of the surface fluctuations leads to an increase in, $R_{a}$ and the unevenness of the skin surface becomes increasingly serious at this time [Fig. 4(h) and Table 2]. Even the most asymmetric frequency distribution also leads to an increase in $R_{sk}$.

According to Table 2, skin samples in the same batch have the same trend, and the difference between the parameter values is small. However, the skin samples between different batches varied due to the differences in the printed cells. Batch 1 had a larger growth rate on days 5 and 13, whereas batch 2 and batch 3 showed a more uniform growth rate. The difference in the Th and $R_{a}$ values of separate batches indicates that there are differences in the degree of AS keratinization between separate batches. Therefore, the adaptive interface extraction algorithm can be used to non-destructively detect the growth regularity of AS during the culture cycle.

By quantifying the thickness and roughness of the normal and abnormal samples on day 7 [Figs. 6(i)–6(p)], it is found that the thickness distribution of the abnormal samples is uneven, indicating that there are thicker or thinner areas, and the roughness has been greatly improved. In the case that the photographic images cannot distinguish the AS sample difference, adaptive interface detection algorithm can correctly distinguish between normal and abnormal samples, which implies that the algorithm in this paper can be used for quality inspection and control of AS samples.

## 6 Conclusion

This paper proposed a real-time, non-invasive, and automated structural parameter analysis method for AS. An adaptive interface detection algorithm was developed to perform the quantification process without human interference in the signals from our OCT setup. The method was first compared with H&E-staining microscopy and demonstrated on standard roughness plates, and then AS quantification during the air–liquid culture cycle was performed. Through continuous monitoring of the AS during the air–liquid culture cycle, the quantitative statistical results show that while the thickness continues to increase, the roughness of the skin decreases first and then increases. Structural parameter quantification of the AS indicates that the change in skin surface roughness is related to the degree of cell keratinization and formation of the corneum, which is further supported by the H&E-staining results. The adaptive interface detection algorithm is also suitable for high-sensitivity, fast detection, and quantification of the interface with layered characteristic tissues and can be used for non-destructive detection of the growth regularity of AS sample thickness and roughness during the culture cycle.

## Code, Data, and Materials Availability

The source files, sample processed data, and pictures of the adaptive interface detection algorithm in this article are all stored in the Github open-source library (https://github.com/MuYuCat/Adaptive-interface-detection-algorithm).
