# Supplementary material for: Automatic quantitative analysis of structure parameters in the growth cycle of artificial skin using optical coherence tomography
Source: J Biomed Opt. 2021 Sep 1;26(9):095001. doi: 10.1117/1.JBO.26.9.095001 (PMC8409365; doi:10.1117/1.JBO.26.9.095001)
Supplement: Supplementary file 1 [file JBO_026_095001_SD001.pdf]

Automatic quantitative analysis of structure parameters in the growth cycle of artificial skin using optical coherence tomography：supplemental document

1. Statical analysis of *Th* and *Ra*

We performed SPSS-based Two-way Repeated Measures Anova [25] on the data of *Th* and *Ra* (see Visualization Table 2). The samples include 3 batches, 15 samples in total.

The results can be concluded that *Th* and *Ra* show significant difference with culture time, which means these structural parameters reveal the culture stage. And *Th* (actually the increase rate of *Th* is used) and *Ra* show no significant difference between batches, which further demonstrates the feasibility of our method as a way for AS quality check in production process. Details in statistical test can be found in below.

1.1 Statistical analysis of Th parameters：

Descriptive Statistics

	Group	Mean	Std. Deviation	N
Day1	1	45.955120	3.9641566	5
	2	37.819400	5.2097939	5
	3	37.081420	1.7359242	5
	Total	40.285313	5.5158313	15
Day5	1	71.205540	2.1759806	5
	2	45.544780	2.7610154	5
	3	47.620500	1.1736953	5
	Total	54.790273	12.2085202	15
Day9	1	78.344560	2.1246612	5
	2	66.242660	3.4389588	5
	3	61.453160	1.1232081	5
	Total	68.680127	7.6916777	15
Day13	1	99.752900	2.5423722	5
	2	80.463760	4.1951680	5
	3	75.646860	.8642345	5
	Total	85.287840	11.1051178	15

Fig. S1. Descriptive statistics

After Mauchly's Test of Sphericity (Fig. S2), it can be seen that sig (p) <0.05, so the data is not spherically symmetric:

Mauchly's Test of Sphericity[a]

Measure: MEASURE_1

					Epsilon[b]		
Within Subjects Effect	Mauchly's W	Approx. Chi-Square	df	Sig.	Greenhouse-Geisser	Huynh-Feldt	Lower-bound
time	.020	41.734	5	.000	.380	.464	.333

Tests the null hypothesis that the error covariance matrix of the orthonormalized transformed dependent variables is proportional to an identity matrix.

a. Design: Intercept + Group
Within Subjects Design: time

b. May be used to adjust the degrees of freedom for the averaged tests of significance. Corrected tests are displayed in the Tests of Within-Subjects Effects table.

Fig. S2. Mauchly's test of sphericity

Therefore, only the test results in the "Multivariate Tests" table are needed. When the values of the various detection methods are different, the relatively stable Roy's Largest Root test results are selected:

Multivariate Tests[a]

Effect		Value	F	Hypothesis df	Error df	Sig.	Partial Eta Squared
time	Pillai's Trace	.959	77.019[b]	3.000	10.000	.000	.959
	Wilks' Lambda	.041	77.019[b]	3.000	10.000	.000	.959
	Hotelling's Trace	23.106	77.019[b]	3.000	10.000	.000	.959
	Roy's Largest Root	23.106	77.019[b]	3.000	10.000	.000	.959
time * Group	Pillai's Trace	1.045	4.008	6.000	22.000	.007	.522
	Wilks' Lambda	.040	13.335[b]	6.000	20.000	.000	.800
	Hotelling's Trace	21.892	32.838	6.000	18.000	.000	.916
	Roy's Largest Root	21.795	79.914[c]	3.000	11.000	.000	.956

a. Design: Intercept + Group
Within Subjects Design: time

b. Exact statistic

c. The statistic is an upper bound on F that yields a lower bound on the significance level.

Fig. S3. Multivariate tests

It can be seen from the Fig.S4. that when the source is time, sig(p) < 0.05, indicating a statistical difference, which proves that the thickness of all samples changes significantly over time.

Tests of Between-Subjects Effects

Measure: MEASURE_1

Transformed Variable: Average

Source	Type III Sum of Squares	df	Mean Square	F	Sig.	Partial Eta Squared
Intercept	144.379	1	144.379	1888.779	.000	.994
Group	.140	2	.070	.913	.427	.132
Error	.917	12	.076			

Fig. S4. Tests of between-subjects effects

It can be seen from the Fig.S5. that when the source is Group, sig(p) = 0.913>0.05 indicates that there is no statistical difference, which proves that there is no significant change between samples in different groups.

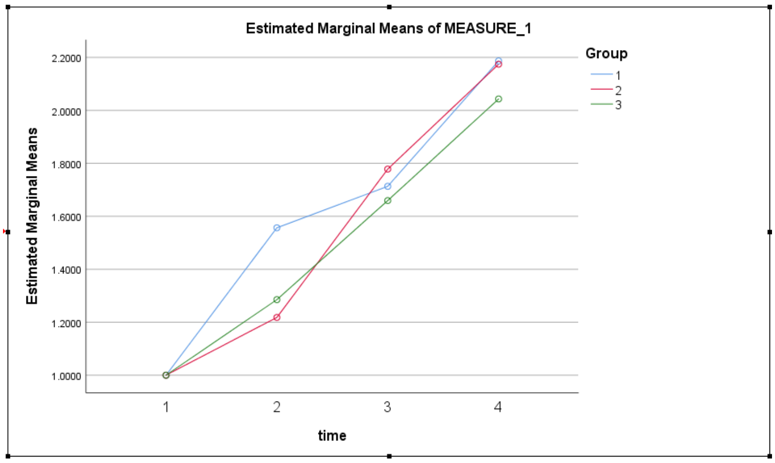

Fig. S5. Trend chart of Th increase rate for different batches

It can be seen from Fig. S5 that there is a consistent trend among samples of different groups, and they all increase with time.

1.2 Statistical analysis of Ra parameters：

Descriptive Statistics

	Group	Mean	Std. Deviation	N
RaDay1	1	3.014140	.0337589	5
	2	3.058760	.3813944	5
	3	2.974220	.1321815	5
	Total	3.015707	.2194440	15
RaDay5	1	1.499720	.1414953	5
	2	1.652960	.2285860	5
	3	1.829680	.1792393	5
	Total	1.660787	.2220425	15
RaDay9	1	1.643980	.1883960	5
	2	1.961760	.2150393	5
	3	2.226000	.3837824	5
	Total	1.943913	.3551008	15
RaDay13	1	2.326960	.4297317	5
	2	2.624740	.1970641	5
	3	2.310640	.2416164	5
	Total	2.420780	.3207348	15

Fig. S6. Descriptive statistics

After Mauchly's Test of Sphericity (Fig. S7), it can be seen that sig (p) = 0.191 > 0.05, so the data is spherically symmetric:

Mauchly's Test of Sphericity[a]

Measure: MEASURE_1

					Epsilon[b]		
Within Subjects Effect	Mauchly's W	Approx. Chi-Square	df	Sig.	Greenhouse-Geisser	Huynh-Feldt	Lower-bound
Time	.499	7.447	5	.191	.762	1.000	.333

Tests the null hypothesis that the error covariance matrix of the orthonormalized transformed dependent variables is proportional to an identity matrix.

a. Design: Intercept + Group
Within Subjects Design: Time

b. May be used to adjust the degrees of freedom for the averaged tests of significance. Corrected tests are displayed in the Tests of Within-Subjects Effects table.

Fig. S7. Mauchly's test of sphericity

Therefore, only the test results of the first row (Sphericity Assumed) in the Tests of Within-Subjects Effects table are required:

Tests of Within-Subjects Effects

Measure: MEASURE_1

Source		Type III Sum of Squares	df	Mean Square	F	Sig.	Partial Eta Squared
Time	Sphericity Assumed	15.839	3	5.280	85.272	.000	.877
	Greenhouse-Geisser	15.839	2.432	6.514	85.272	.000	.877
	Huynh-Feldt	15.839	3.000	5.280	85.272	.000	.877
	Lower-bound	15.839	1.000	15.839	85.272	.000	.877
Time * Group	Sphericity Assumed	.871	6	.145	2.344	.052	.281
	Greenhouse-Geisser	.871	4.863	.179	2.344	.068	.281
	Huynh-Feldt	.871	6.000	.145	2.344	.052	.281
	Lower-bound	.871	2.000	.435	2.344	.138	.281
Error(Time)	Sphericity Assumed	2.229	36	.062			
	Greenhouse-Geisser	2.229	29.179	.076			
	Huynh-Feldt	2.229	36.000	.062			
	Lower-bound	2.229	12.000	.186			

Fig. S8. Tests of within-subjects effects

It can be seen from the Fig.S9. that when the source is time, sig (p) <0.05, indicates that the roughness of all samples has changed significantly over time.

Tests of Between-Subjects Effects

Measure: MEASURE_1

Transformed Variable: Average

Source	Type III Sum of Squares	df	Mean Square	F	Sig.	Partial Eta Squared
Intercept	33.931	1	33.931	2821.617	.000	.996
Group	.075	2	.037	3.107	.082	.341
Error	.144	12	.012			

Fig. S9. Tests of between-subjects effects

It can be seen from Fig.S10 that when the source is Group, sig(p) = 0.082 > 0.05 indicates that there is no statistical difference, which proves that there is no significant change between samples in different groups.

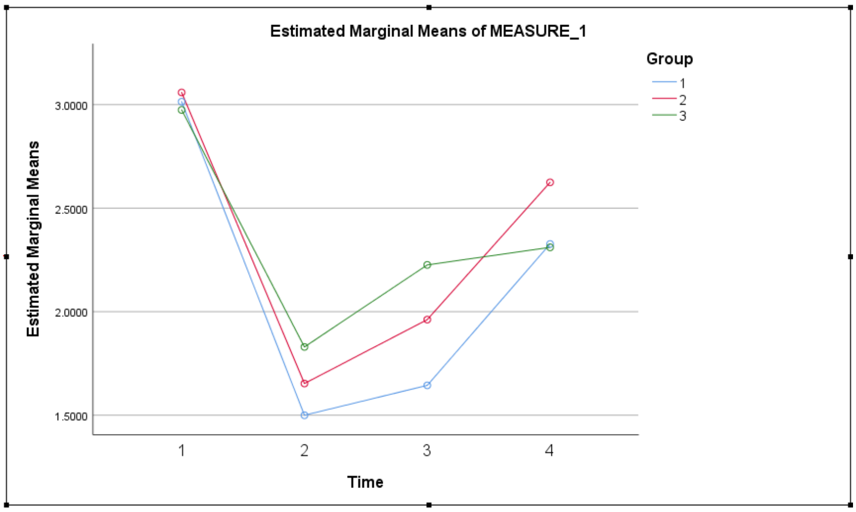

Fig.S11. Trend chart of Ra for different batches

It can be seen from Fig.S11 that there is a consistent trend among samples of different groups, all showing a trend of first decreasing and then increasing, and reach the maximum on Day 5.

In order to verify the feasibility of our method, we performed repeated measures multi-factor analysis of variance to statistically analyze the *Ra* and *Th* data (see Visualization Table 2). Considering the difference in thickness at the starting point, the increase rate of *Th* is adopted for analysis. Statistical analysis with time is performed, and we obtain F_Th_rate(3,10)=77.019 (P=3.2677*10-7<0.05) and F_Ra(3,36)= 85.272 (P=2.0111*10-16<0.05). The results indicate that *Th* and *Ra* have significant statistical differences with time, which shows that the culture stage can distinguished be by such parameter monitoring. The results of statistical analysis between batches show that F_Th_rate(2,12) = 0.913 (P=0.427>0.05) and F_Ra(2,12) = 3.107 (P=0.082>0.05), which means the value of *Ra* and the increase rate of *Th* show no significant statistical difference between batches.
